# “Is There Room for Both Loves?”: The Experience of Couplehood Among Women Living With a Widower With Young Children

**DOI:** 10.3389/fpsyg.2022.870467

**Published:** 2022-04-25

**Authors:** Talia Peichich-Aizen, Dorit Segal-Engelchin

**Affiliations:** Spitzer Department of Social Work, Ben-Gurion University of the Negev, Be’er Sheva, Israel

**Keywords:** second couplehood, widowhood, deceased wife, widowers, women

## Abstract

Very few studies to date have explored the couplehood relationship in blended families with young children created after widowhood. This study sought to add to our knowledge of this issue by examining the couplehood experience of women who started a family with a widower with young children, with no children of their own. Semi-structured interviews were conducted with 30 Israeli women aged 32–78 years. The findings indicate that many participants feel that the deceased wife continues to be present in their partner’s life and that she is an integral part of their couplehood relationship. The participants described two subsystems existing alongside the couple subsystem with their partner, namely, the partner’s spousal subsystem with his first wife; and a triadic subsystem consisting of the woman, her partner, and his deceased wife. The perceived presence of the first wife raised poignant questions concerning the place of the two women in the partner’s life. Participants’ narratives revealed the dissonance between understanding and acknowledging their partner’s continuing bond with and affection for his deceased wife on the one hand, and recognition of his love for them on the other hand. The findings shed light on the complexity inherent in a couple relationship with a widower and may assist professionals who provide support to blended families in understanding the unique challenges faced by these women. This would enable them to tailor their counseling and the therapeutic process to the particular needs of these women.

## 1. Introduction

Women who start a family with a widower with young children are forced to contend with a unique type of couplehood (Hickey, 1998; Lavy, 2015). Numerous studies have considered the couple relationship in blended families after divorce (Adler-Baeder and Higginbotham, 2004; Mirecki et al., 2013; Shafer et al., 2013; Zionov, 2015), and growing research attention is being paid to remarriage after the loss of a spouse when the children have already left home (Davidson, 2002; Carr, 2004; De Jong Gierveld, 2004; Carr and Boerner, 2013; Osmani et al., 2018; Ayuso, 2019). However, only a few studies have focused on couplehood in blended families after widowhood when the children are still at home (Kissman and Dane, 2001; Bishop and Cain, 2003; Baker, 2014; Bokek-Cohen, 2014). Moreover, a review of existing studies shows that less is known about the experience of partners of widowed individuals. Most studies do not distinguish between widows and widowers, disregarding any possible differences between the two family models (Moss and Moss, 1996; Grinwald and Shabat, 1997; Kissman and Dane, 2001; Brimhall and Engblom-Deglmann, 2011). The couplehood experience of women living with widowers with young children has received less attention in studies (Hickey, 1998; Lavy, 2015) that are related to various aspects of their lives. This study sought to add to our limited knowledge of these women by examining the way in which they experience their couplehood.

### Couplehood in Blended Families

Blended families, which are becoming increasingly common (Ben-Yehoshua and Sabar, 2012), pose unique challenges for the couple. Unlike the relationship that develops in a first marriage, a second marriage begins in the presence of children from the former relationship (Pacey, 2005; Ben-Yehoshua and Sabar, 2012) and the presence, whether physical or otherwise, of the previous spouse (Ganong and Coleman, 2004; Martin-Uzzi and Duval-Tsioles, 2013). This reality is likely to complicate the building of a couple’s relationship, as the partners do not have the opportunity to spend time in a couple space, which allows for the development of intimacy, mutual understanding, and sharing, without the presence of a third party (Ganong and Coleman, 2004; Pacey, 2005). Furthermore, the new couple’s relationship takes shape against the background of a previous family history made up of rituals, values, and shared meanings that have yet to be constructed by the new couple (Dupuis, 2007). This may lead to tension between the couple, as the partner entering the existing family structure may feel they do not belong (Ganong and Coleman, 2004). In addition, the literature indicates that second marriage, whether after divorce or the death of a spouse, is created in the shadow of the loss of the former relationship (Pacey, 2005). Nevertheless, the different reasons for the dissolution of that relationship necessitate different types of coping. While divorce results from the desire of at least one of the two sides to end the relationship, which is therefore perceived as a failed marriage (Martin-Uzzi and Duval-Tsioles, 2013), widowhood is involuntary (Bokek-Cohen, 2014). The limited literature that focuses on couplehood after the death of a spouse suggests that the experience of loss is a central factor in understanding the new couplehood and presents the couple with unique difficulties (Brimhall and Engblom-Deglmann, 2011).

### Couplehood After the Loss of a Spouse

A review of the literature on couplehood following the death of a spouse indicates that loss is an integral part of the new romantic relationship and that this relationship does not replace the previous one but develops alongside it (Moss and Moss, 1996; Silverman and Klass, 1996; Grinwald and Shabat, 1997; Brimhall and Engblom-Deglmann, 2011). For most of the twentieth century, ongoing ties to a deceased person were regarded as an indicator of pathological grief (Klass, 2006). This perspective was grounded in Freud’s (1917/2009) “Mourning and Melancholia” in which he argued that successful adaptation to loss requires psychological separation from the deceased and relinquishing the bond with them to complete the grieving process. Klass et al. (2014) were the first to introduce the notion of the “continuing bonds paradigm” (Florczak and Lockie, 2019), describing the connection with a deceased loved one as normal rather than pathological. This approach also informed examinations of the loss of a spouse (Bauer and Bonanno, 2001; Lowe and McClement, 2011; Bokek-Cohen, 2014), which investigated the effect of continuing bonds on adaptation and coping with loss. Some found a positive effect. Thus, Lowe and McClement (2011) reported that widows’ continuing bond with their deceased husbands strengthened them and helped them to maintain the continuity of their identity. Similarly, Bauer and Bonanno (2001) found that the ongoing connection played an important role in creating a sense of meaning and personal continuity after the loss. Other studies, however, reported a negative effect of continuing bonds on adaptation and coping (Klass, 2006; Root and Exline, 2014; Florczak and Lockie, 2019).

The complexity of the influence of a continuing bond also stands out in studies examining its effect on couplehood with a widow or widower. Several studies indicated that coping with a partner’s continuing bond with a deceased spouse is not an easy matter (Moss and Moss, 1996; Silverman and Klass, 1996; Grinwald and Shabat, 1997; Brimhall and Engblom-Deglmann, 2011; Bokek-Cohen, 2014). Kissman and Dane (2001) presented a case study of a widower who remarried a divorced woman and noted that the widower’s ongoing connection to his late wife alongside the development of the new couplehood led to a triadic relationship that included the deceased woman, thereby interfering in the creation of the second relationship. The complex ramifications of this triadic relationship can also be seen in the study by Bokek-Cohen (2014), who examined the couple experience of young widows and their spouses. The new partners reported that their wives’ continuing bond with their previous husband gave them the sense that she was married to two men. Although she loved them and was committed to them, she also continued to love and be committed to her deceased husband. Similarly, Moss and Moss (1981, 1996) examined couplehood after the loss of a spouse among older couples and found that the partner of the widow or widower was coping with the fear that their spouse’s commitment to their first marriage came at the cost of their commitment to them.

A further difficulty for the new spouse relates to the tendency of widows and widowers to idealize their previous partner. Indeed, the literature on loss, in general, indicates the tendency to eulogize the dead (Hayes, 2016). O’Rourke (2004), who examined the adjustment to bereavement of older widows, found that this tendency facilitates better adaptation. Nevertheless, idealizing a deceased spouse may make it difficult for a new partner to feel appreciated (Grinwald and Shabat, 1997; Brimhall and Engblom-Deglmann, 2011). In addition, it may cause the new partner to draw a comparison between the two relationships (Brimhall and Engblom-Deglmann, 2011), potentially leading to a sense of insecurity (Moss and Moss, 1981; Brimhall and Engblom-Deglmann, 2011).

To conclude, a review of the existing literature reveals that few studies have considered couplehood with a widow or widower from the perspective of the new partner. Furthermore, to the best of our knowledge, no previous study has related specifically to women who started a family with a widower with young children, with no children of their own. This study sought to expand our knowledge of this population by examining their experience of a couplehood that develops against the background of the loss of their partner’s first wife.

## 2. Materials and Methods

The study employed a constructivist-qualitative paradigm using the principles of Charmaz’s constructivist grounded theory (Charmaz, 2006, 2008, 2009).

### 2.1. Participants

The sample consisted of 30 women who had been in a family relationship with a widower with children. The eligibility criteria for inclusion in the study were that they were in a relationship with their widowed partner for at least 1 year; that at the start of the relationship, at least one of their partner’s children was under the age of 18 years; and that they had no biological children of her own. Several methods were used to recruit participants: (1) An appeal to Internet support groups dealing with widowhood; (2) a call for participants posted on Facebook; (3) a notice calling for participants hung on bulletin boards throughout the campus of the researchers’ university; and (4) the snowball method (Noy, 2008), whereby women who agreed to take part in the study were asked to refer to the researchers other women with whom they were acquainted who met the criteria.

All the participants had been born in Israel and all were Jewish, save for one Muslim Arab woman. Ten defined themselves as religious. Age at the time of the interview ranged from 32 to 78 years. In terms of education, 24 had an academic degree, five had post-secondary education, and 1 had a high-school diploma. For almost all the women, this was their first familial relationship, with only two being divorced. All, except one, were married to their partner. Years in the relationship ranged from 1.5 to 39 years, and the age of the partner’s children at the start of the relationship ranged from 1 to 18 years. In only two cases did the man have older children as well.

### 2.2. Interviews

Semi-structured interviews were conducted with the participants. The first six interviews centered around a research question concerning the woman’s new role as mother. In describing their experience of motherhood, all six participants, with no exceptions, also referred at length to issues they were not specifically asked about, including the decision to enter a relationship with a widower with young children; their place, and that of the deceased wife, in their partner’s life; and the sense of foreignness in the family home. Consequently, the research question in the following interviews was expanded to relate to the woman’s overall experience and not solely her experience of becoming a mother. In the first section of these interviews, the participants were asked to speak freely about their experience as women in a couple relationship with a widower with young children. In the second section, they were presented with specific questions, outlined in the interview guide, which were similarly expanded as part of the circular process of grounded theory (Strauss and Corbin, 1990), in which data collection and analysis are conducted in parallel.

The time and place of the interviews were chosen by the participants, and they were conducted by the first author. Each lasted between 90 min and 3 h. The interviews were recorded and fully transcribed with the participants’ permission.

Content analysis was performed in the three stages suggested by Strauss and Corbin (1990), namely, open coding, axial coding, and selective coding. In open coding, the data are broken down, conceptualized, and categorized. In this study, this process revealed contents not included in the research question initially framed, which related solely to the experience of motherhood. As noted above, this led to the understanding that motherhood is only one part of the women’s multifaceted experience. As a result, after the first six interviews, the research question was expanded to relate to the participants’ overall experience, with the questions in the interview guide aimed at allowing for a broader observation of their lives on various levels. Each of the contents that emerged from the following 24 interviews was subjected to open coding, making it possible to identify a variety of categories.

Axial coding, during which connections are drawn between the categories and subcategories (Strauss and Corbin, 1990), revealed three main categories, namely, the experience of home, the motherhood experience, and the couplehood experience. This study focuses on the experience of couplehood.

Selective coding identified the processes and characteristics of each of the three main categories. With respect to the experience of couplehood, several subjects that played an important role in shaping this experience were identified. The analysis was directed to understanding the connection and hierarchy between them as reflected in the participants’ narratives. Three major subcategories of the couplehood experience were identified, namely, the presence of the first wife in the life of the partner and family, the factors presencing the deceased woman, and the new woman’s place in her partner’s life.

### 2.3. Ethical Considerations

The study was approved by the Ethics Committee of the authors’ university. The aims and procedure of the study were explained to each participant, who then signed an informed consent form. To protect participants’ confidentiality, pseudonyms were used.

## 3. Results

### 3.1. The Presence of the First Wife in the Life of the Partner and Family

The overwhelming majority of the participants stated that the fact that their partner was a widower impacted the new couplehood. A dominant theme, iterated in numerous interviews, was the sense that the deceased wife continued to be present in the partner’s life and was still an integral part of family and couple relationships:

*I don’t believe there’s a day that he doesn’t think of her (Michal*^1^ *).*

*I’m certain he talks to her. I’m sure of it. He would never tell me, not because he’s hiding anything but because it’s not in his nature to share. But I’m 100% certain he has conversations with her. I’m sure (Shiraz).*

Some participants attributed the first wife’s presence to the tragic manner in which their partner’s relationship with her ended. As they described it, death terminated a relationship that did not end voluntarily, and they noted the difference between a marriage that ended in divorce and one that was cut short by the death of a spouse:

*He was in a relationship with a woman for 30 years, and they didn’t want to end it. It ended because of an illness*… *In the case of a widower, there was a relationship, and that relationship ended in a way no one chose*… *I had that feeling, that the relationship wasn’t over. It’s a very different situation from divorce. In a divorce, there’s definition. There’s a breakdown, a decree, the relationship is over! There’s a beginning and an end. Not in widowhood. There’s an involuntary separation. It’s different (Na’ama).*

The presence of the first wife was manifested in varying intensity in the course of the couple’s relationship. Her continued presence aroused a broad range of emotions and difficult questions regarding the woman’s own place in her partner’s life. The interviewees related to three primary factors presencing the deceased woman in the couple’s relationship, namely, their own need to learn about her, their partner’s need to preserve her memory, and family events.

### 3.2. Factors Presencing the Deceased Woman in the Couple Relationship

#### 3.2.1. The Woman’s Need to Learn About the First Wife

Most of the participants expressed a desire to learn about the deceased woman through stories and memories. As Ya’ara put it:

*I felt I wanted to see more and more pictures of her, especially with the girls*…*to know how it once was*…*what his [her partner] family was like, how the four of them were together, how it was when they just met and how it was when they were a young couple with one daughter and then with another daughter.*

The interviews revealed a variety of reasons for this need. For example, in the case of Orly, it was related to the fact that she was raising the woman’s children, and it was, therefore, important for her to know as much as she could about their mother:

*I said, it can’t be that everyone knows her so well except for me. That’s an impossible situation*… *Like, here I am raising her children*…*and I felt like*…*like*…*I have to get to know her as well as I can.*

Galit attributed her need to learn about the deceased woman to her desire to be part of her partner’s past:

*Some of the memories, I want to be part of everything he brings with him. He brings it with him in the present and in the past and in the future. And I want to be part of the past he had. It’s very significant for the whole personality I married. That’s very important to me.*

For most of the participants who expressed their need to learn about the deceased woman, this need was present at the very start of the relationship:

*At the beginning I remember I asked him a million questions about her*… *I told him “I’m curious. I want to know who she was”*… *She’s part of his life, of the children’s life, and this is my family. So from the beginning I didn’t only get to know him and the children, I got to know her too (Orly).*

Other women indicated that the need evolved gradually, appearing at a later stage in the couple’s relationship:

*I remember it took time until*… *I preferred not to know what she looked like. At the beginning it really really scared me. I wanted to stay in my bubble. I knew there was a woman who died but I didn’t*… *Gal [her partner] wanted very much for me to see pictures of her*… *I don’t know. I was afraid to look*… *I don’t remember what scared me, but I remember I wanted to put it off while we were inside the bubble of a beginning (Ya’ara).*

Alongside the need to learn about their partner’s first wife through family photos and anecdotes, many participants described the difficult emotions these memories aroused. It was particularly hard for them to deal with memories relating to the previous couple’s relationship. For example, they were more comfortable looking at family pictures or photos of the mother and her children alone, but pictures presenting their partner’s relationship with his first wife were hard for them to handle. For example, one woman spoke of her difficulty coping with the preservation of the memory of her partner’s relationship with his deceased wife in the home. She reported that after they renovated the house, she put back photos of the first wife alone or with her daughter, but not the romantic picture of the couple:

*When you come into our house*…*um*…*even before we met there were two pictures of Shani [the first wife] with Noa [her daughter], one in the living room and one in the dinette, and there were similar photos on the refrigerator as well as a lovely one of the three of them together. There was also a photo of him with Shani, a very romantic one. They were kissing or something like that. After the renovation*…*the pictures were put back in place but now it wasn’t exactly the same place*…*and the photo of the romantic kiss didn’t go back where it was (Shachar).*

The participants also spoke at length about the difficulty of dealing with memories in which the deceased woman was idealized by their partner. Many stated that their partner tended to describe his first wife as the perfect woman, highlighting her virtues. For example, Lotem noted that the idealization of the previous wife made her undervalue herself:

*This practice, that when someone dies they become some sort of*…*like an angel on Earth. What human being is so perfect and amazing? And*…*it’s like you’re taking their place*… *You’re like*…*stepping into their shoes, and*…*it’s like*…*it’s a heavy burden, like how can I*… *At the beginning it made me very emotional, at the beginning I didn’t give myself much credit (Lotem).*

Sima described how the idealization of the deceased wife placed her in a disadvantaged position:

*But you can’t say anything bad about her because she died and she’s up there on some sort of pedestal like a saint. And I always, I get irritated and angry. I’m a human being and I can’t compare to her. That’s the price you have to pay when you’re in a relationship with a widower*… *As if I expected him, you know, to say something about his wife that was annoying, but he’s never said anything negative about her (Sima).*

While most of the interviewees referred to their need to learn about the first wife at some stage in the relationship, others expressed reservations about doing so. Na’ama’s reservations, for instance, stemmed from the fear that preserving the past would place obstacles in the path of the new relationship:

*I didn’t want to hear about his first wife, what happened and how it was. I worked on the assumption that if he was choosing to begin a relationship now, then you put the past aside and start a new relationship. It’s the same as my not talking about my exes and what it was like with them. It’s not relevant and not appropriate (Na’ama).*

#### 3.2.2. The Partner’s Need to Preserve the Memory of His First Wife

The participants regarded their partner’s need to preserve the memory of the first wife as the major reason for her presence in the couple’s relationship and evidence of his continuing bond with her. The large majority of women reported that their partners exhibited this need at varying intensity in different stages of the relationship. It was expressed primarily in two ways, namely, keeping items associated with the deceased wife and commemoration of her.

##### 3.2.2.1. Keeping Items Associated With the Deceased Wife

The interviews revealed the widowers’ need to hold on to items belonging to their first wife to preserve her memory. For example, one participant spoke of a drawer in which her partner kept the deceased woman’s personal items. The drawer was perceived as the partner’s private space:

*He has a drawer I’ve never opened. There are a few of her [his first wife’s] things in there, her identity card, a framed picture. I don’t know, I never opened it. I have no interest in it. It’s not mine*… *It’s in a three drawer dresser. I know what’s in the top drawer, because he puts his wallet and a flashlight in it*… *But that drawer I don’t open*… *I don’t know. It’s his private territory. I don’t touch it (Mira).*

Holding on to items symbolic of the first wife, and letting go of these items, appears to be part of a dynamic process. Most of the participants related that at the start of their relationship, their partner had difficulty letting go of these items, but it became easier over time. For example, one woman noted that only after several years of marriage did her husband decide to give away the deceased woman’s clothes. Other participants spoke of their partner’s wedding ring from his first marriage, which they regarded as symbolizing the previous relationship and presencing the first wife in their couplehood. As Galit put it:

*The ring makes it tangible to me that he had a relationship before me. That he loved somebody before me. I know that, I entered the relationship knowing that. It’s a fact. But there’s something in physical objects that makes it very concrete.*

She went on to describe the process her partner underwent with respect to the ring:

*Today it’s nothing. I remember a little something from 7 years ago*… *After he took off the ring, it was in his drawer*… *We talked about it, He moved the ring to a box that actually belongs to the girls and it’s still in the house. But, like, it’s not in his drawer. Like, it’s in a box of things he put away, that he wants to keep for the girls (Galit).*

The participants experienced taking the ring off as an act symbolizing the end of the first marriage and the process of letting go of the previous couplehood. When this did not occur, the women were apt to feel insecure in their relationship. This is apparent from the words of Lotem, who had been in a relationship for 5 years, but felt that her partner’s wedding ring from his first marriage, which he continued to wear, prevented the creation of a new emotional couple space:

*He still wears the wedding ring*… *He hasn’t entirely let go [of his first marriage], as if it’s still there somehow*… *She [the couple therapist] confronted him with it*…*with the fact that he still has the wedding ring on his finger*…*about letting go and about*…*about the fact that I*…*don’t feel*…*like I don’t feel secure enough in my place. I feel as if*…*it’s always there under the surface, in a very very sensitive place, very charged, and it has to do with a process of letting go that he has to undergo with himself. It can and should be a part of life, but at*…*a level of coming to terms with it more.*

Later in the interview, Lotem stated:

*I feel inside that*…*he still doesn’t entirely accept me. It will happen when he releases something*… *He doesn’t have to let it all go, but*… *he has to free himself from something in order to let himself accept me too*… *I don’t feel secure enough in the place I’m in.*

##### 3.2.2.2. Commemorating the Deceased Wife

The partner’s need to preserve the memory of his first wife is also expressed in various forms of commemoration, such as preparing a memorial book, organizing a walk in her name, keeping pictures of her in the home, or visiting her grave on the anniversary of her death. Most participants reported that the acts of commemoration decreased over time, with some attributing this decline to the strengthening of the new couple’s relationship:

*In a certain way I think he was a widower by definition. He was still preoccupied by memories of her and preparing her book and his preoccupation with it helped him process it, process his grief*… *He tried to sell her artworks and things like that, and it seemed to me it was also part of the processing. I think as soon as he decided he was ready to get married, it all subsided. This preoccupation wasn’t there anymore (Vered).*

In contrast, several participants noted that their partner continued to be absorbed in preserving the memory of his first wife even after he was in a new relationship. For example, in the case of Hannah, this went on for many years into the marriage, arousing in her a sense of discomfort and of being in competition with the deceased woman:

*I think somehow when we got married I expected him to put it aside*… *I said to him. “Why do you have to involve yourself in other things associated with your dead wife? There’s a memorial service, you put out a book, why do you have to reach out to other niches related to her? Why do you go to those places? I’m your wife!”*… *It’s like he was drawn there*… *He didn’t let go. He says, “What do you mean? I have to do it.”*

##### 3.2.2.3. Family Events

The participants spoke at length of the way in which family events, such as memorial services and family celebrations, especially those of their non-biological children, presenced the first wife in the life of the couple and the family. Eden, for instance, described how she felt her strong presence in her partner’s life at these times:

*I have no doubt that she pops up in his world and she’s there with him, doesn’t just pop up, but pops up and stays there for a visit. Certainly before special events, certainly when the children start to get married. At the bar mitzva* ^2^ *and bat mitzva* ^3^ *we celebrated, it was obvious to me that he missed her much more, and he needed her to be there much more, and he communicated with her more*… *I know he also writes to her sometimes.*

The deceased woman’s birthday was also commemorated in different ways by several of the participants’ families. One interviewee noted that the whole family got together every year on her birthday:

*On Roni’s [the first wife’s] birthday it’s become a tradition for the whole family to come to our house on her birthday. We try to make it the precise day, but if it doesn’t work out then the day before or the day after everyone gets together at our house*… *It’s a very strange occasion because there’s a need for them to be together, but it’s not really a birthday party*…*and they hardly talk about her. It’s not like they all meet and “let’s talk about Roni,” but she’s very present there*… *So it’s a strange occasion but they keep doing it because it’s become sort of a tradition (Shachar).*

Another participant said that commemoration of the deceased wife’s birthday included a visit to her grave with her partner:

*The whole week leading up to his first wife’s birthday he’s not there*… *What made it even harder was that I had just gotten pregnant and I was all excited*…*and we were looking for a house, and I thought how can you be here and there at the same time. He has this amazing ability I can’t understand*… *But I feel like the guy is sort of*…*fading out*… *And I find myself in a surrealistic world going with my husband to buy balloons and flowers for his dead first wife that he doesn’t buy for me because he doesn’t like*… *I just didn’t know what to do with myself that day (Michal).*

Another family event at which the presence of the deceased woman is particularly strong is her annual memorial service. Eden noted that on the days before the service, her partner would refer to her more frequently:

*Before the memorial service, he’d mention her more often in our conversations and at Friday night dinner he’d say to the kids, “I still think about Mom, I think about her every day. She’s always with us.”*

Dikla described how difficult it was for her when her partner chose to praise his first wife at the service by singing “Woman of Valor.” It made her feel that his love for his first wife was greater than his love for her:

*At the memorial service, all of a sudden he said: “Okay, so I want to devote this song to my wife, may she rest in peace,” and started to sing the song, “Woman of Valor.” It felt like a slap on the face. That’s exactly what I felt, and I had nowhere to escape to. It was a small house, there were a lot of people, and I had nowhere to run to*… *After that I simply couldn’t talk to him. The next day, someone called me, a widow who had married a single man, to give me her support. I said to her, “I don’t know if there’s any way back from here, how he could, right before my eyes, sing ‘Woman of Valor’ to his first wife as if he was in the throes of longing and yearning.” And then she said, “Think of it differently. It doesn’t necessarily mean he doesn’t appreciate your virtues”*… *I wasn’t capable, because I felt like he was there and he wasn’t here. I felt he loved what he had had, and he loved her now as well.*

Shachar related that while in the past the strong presence of the first wife at the annual memorial service had undermined her confidence in her relationship, things had changed over time:

*Then, at the service one year, the things he wrote*… *He wrote about how he finds himself consulting with his first wife, telling her things, passing by her picture and telling her*…*thinking about what she would say*… *um*…*like some sort of intimacy he has with her that I’m not part of*… *Again, I think today we’re in a different place than five years, four years, ago*…*um*…*because my presence and the existence of our family and of Guy [her husband] and me as a couple are already so established that*…*it’s not there. Today it isn’t a threatening space, but I remember I read it and I said, Wow.*

Nevertheless, most of the participants stated that the presence of the first wife is supposed to be dominant at the memorial service, and they actually choose to play down their own presence at the event. Some opted not to go to the gravesite, as they believed the day should be devoted to their partner and his first wife, and they had no place there. Even those who did go stressed their need to minimize their presence:

*It’s hard for me when I’m mentioned at the memorial service. It isn’t appropriate. Those aren’t days to talk about me*… *It’s not the time to praise me or show me respect*… *It’s hard for me, when they refer to me during the service. It’s her day alone, her place alone (Deborah).*

### 3.3. The New Woman’s Place in Her Partner’s Life

The fact that the new couplehood takes shape against the background of the partner’s first marriage and the continued presence of his first wife raises questions as to the place of the new woman in his life. The interviews indicate two issues in particular, namely, the partner’s preference, that is, “her or me?”; and “is there room for both loves?”

#### 3.3.1. “Her or Me?”

The participants’ need to contend with the question of their place and the place of the new relationship in their partner’s life raised the issue of which of the women the partner preferred. They spoke of wondering, if, given the opportunity, their partner would choose themselves or his first wife. For example, Orly described how difficult it was for her when her partner shared a recurring dream in which he had to choose between the two women:

*He’d wake up from a dream at night and tell me he had dreamt she’d come back and that I was there and she was there, and he had to decide. He’d describe it to me [she laughs]. He didn’t keep anything to himself. It was hard for me to get up in the morning after he told me about his dream. It would stay with me*… *It was hard knowing that if she [his first wife] came back*…*it wasn’t a given that he’d choose me again. That’s hard. Or maybe he would, I mean, it’s not clear, not clear*…*but it*…*it weighed on me.*

Another participant similarly noted that the question of her partner’s preference echoed in her mind and remained unanswered:

*Sometimes I’m troubled by the question, what would happen if we were both there, who would he choose? I think he would choose me, of course, no question about it [she laughs], but he might choose her, because she’s different (Malka).*

This issue arose particularly when the couple’s relationship was not going smoothly:

*Look, we don’t fight a lot, you know, real serious fights. It doesn’t happen often, but when it does, sometimes it seems to me he’s saying to himself, “I wish Snunit [his first wife] was here. It was much nicer with her.” I’m not sure he says that. I could ask him. He’d undoubtedly say no. But sometimes it seems to me*…*let’s say, if I were him I’d say it, What do I need this for?! She’s so annoying, it was so great with my wife. What do I need this for? It’s too bad she died (Ya’ara).*

For most of the interviewees, the question of their partner’s preference remained open. Several participants, however, expressed the feeling that they would always be their partner’s second choice:

*I’ll always be the second wife*…*um*…*It’s good and it’s bad. Like, he compares us, he doesn’t compare us*… *He didn’t get a divorce, but there was a woman there before*… *When it comes down to it, it ended with a lot of love (Michal).*

*I told him, “I know that feeling*…*like there’s a multi-storied building and like she’s on the first floor, and the first floor is important. I feel that I am the second floor*…*” I’m not complaining, but that’s how it is. I’m not the first (Dikla).*

#### 3.3.2. “Is There Room for Both Loves?”

Another theme that emerged from the interviews was the question of whether there was room for two loves in the partner’s life. Most of the participants stated that their partner continued to love his first wife. Thus, Ilana described a letter he read out at his deceased wife’s memorial service:

*He wrote to her on the anniversary of her death and read it out at the memorial service: “There is a love you have for people who are gone and will never return, people a large part of whom remain deep in your heart—a love that will never end.”*

Later in the interview, she referred to the confusion aroused by her partner’s simultaneous love for his first wife and for her:

*I’m a little confused*… *He loves her and me too*…*But the word “love” should belong to one woman. It’s a complexity I live with (Ilana).*

Other participants also expressed the difficulty of coming to terms with this duality:

*Suppose I say he does feel a connection and love for his first wife, then it’s like I don’t feel loved enough*… *Sometimes yes, sometimes no. I feel very appreciated, very much so, but love*…*? When he also loves her? (Malka).*

*For many years it was hard for me to accept that he could keep telling me he loves me, because how can you say you love me at all when I know you worshipped her and loved her very much, and*…*all you had together (Rotem).*

*It can arouse, um, jealousy, thinking, “Wait a minute, does he really love me or does he love her?” Like, “Is he with her or is he with me?” It has to do more with an imaginary relationship. Like, it matters very much where the greater weight is (Galit).*

These quotes shed light on the complexity inherent in a relationship with a widower, revealing the difficult questions that the dual love poses for the new woman. The participants referred to viewing this complexity from a variety of perspectives that helped them accept the situation. Orly, for instance, attempted to give it a logical explanation:

*The feeling, but it’s a feeling that stayed with me, that he loves her very much. I’d ask him all the time if he misses her*… *At the beginning it was strange. But at some point it became*…*like, I said, I explained it to myself. It’s logical, it makes sense! The guy loved somebody very much and she disappeared on him! Of course he loves her. Like, we love people we are separated from against our will. It’s not over, and it will go on*…*um*…*and that’s okay. I mean, it’s, it’s*…*it’s right, it’s logical. Is it good? I don’t know if it’s good.*

Although they had difficulty coming to terms with the fact that their partner still loves his first wife, several participants also noted that this love poses no real threat to them for a number of reasons:

*There are things about a relationship with a widower that are comforting, in inverted commas. She can never turn up again (Orly).*

*She was there until a certain point in time and I’m there from another point in time, and there’s no overlap. She doesn’t threaten to enter my time frame and I don’t threaten to go back to hers. One was then and one is now (Yaara).*

*One of my exes always talked about his previous girlfriend. Sharon [her partner] doesn’t do that at all. To his credit, I’ve never heard a single word of comparison, and*…*um*…*but I live with the knowledge that the heart is divided into different parts (Michal).*

The couple dynamics was considered by the women the most important factor in resolving the ambiguity inherent in the dual love. For some women, the insecurity regarding their place in their partner’s life gave rise to a need for positive reinforcements from him to prove his love for them:

*We’re very much together, really going through it together. Um*…*he always conveys trust and a very deep love, always reminds me of it. I think it’s very very meaningful, um, like giving me the*…*he helps me maintain a sense of security*… *I feel that if I didn’t have a sense of security, it would be very hard to be in this situation, like entering a place, a space I may not fill, and maybe I don’t do enough, and maybe I’m not loved enough, and maybe and maybe and maybe*… *I think he plays a very important role here in strengthening it, that place, telling me he loves me. I think it’s really important (Galit).*

*I felt as if he made room for me, he absolutely made room for me*… *Certainly in the emotional space, and also in the general space. I remember saying to him even before we got married, I said, “After the wedding when we’re walking down the street you’ll hold my hand. You can also kiss me in public. Now, that wasn’t something they [her husband and his first wife] did before. I said, “I don’t care! It’s allowed by religious law. I want it. I need that proof, I need that public expression. I need the children to see it, to see that you love me.” What can I say, it was important to me. I think he learned to like it in time. To acknowledge it (Eden).*

These words demonstrate how the perceived presence of the deceased woman impacted participants’ couplehood experience. Indeed, many of the participants reported feeling that they had entered a triadic relationship that included the first wife:

*Every time*…*we came to certain points in life*…*um*…*or special events*…*I always felt like Ofra [the first wife] was sitting here [she points to one shoulder] and Arik [her partner] was sitting here [she points to her other shoulder] and I have to carry all of us into the event (Orly).*

*Like*…*um, what I felt, that we’re almost three, that she’s there in our space*… *She’s there in the context of a partner*…*with all the memories surrounding that space, and go find your place*… *I had to find my place*… *Now, for me that was very hard (Malka).*

Rakefet felt that the first wife was even present at intimate moments:

*It bothered me, just the thought that he was looking at her picture and thinking of her and I’m sitting next to him. The thought went through my mind that he’s hugging me and kissing me and thinking of her*…*as if she was with us.*

The triadic relationship created a dissonance the women had to contend with, as reflected in the following quote:

*In parallel to me, he leads another life with Roni [the first wife], as if there’s a life with another woman, and I can’t complain, he’s not cheating on me [she smiles]. It’s not cheating, but what he had there I can’t provide and what he has with me he can’t get there (Shachar)*,

## 4. Discussion

This study investigated the couplehood experience of women who started a family with a widower with young children, with no children of their own. Similar to previous studies of couplehood with a widower (Moss and Moss, 1981; Grinwald and Shabat, 1997; Brimhall and Engblom-Deglmann, 2011; Klass et al., 2014), the results show that most of the participants felt that the deceased first wife was an integral part of the family and their relationship with their partner. Many of the interviewees noted that the presence of the first wife in these relationships stemmed both from their own need to learn about her and their partner’s need to preserve her memory. Her presence aroused conflicts and questions about their own place in the couple relationship.

The concept of “subsystems,” a fundamental component of the model of Structural Family Therapy (SFT) developed by Minuchin (1974), may provide a theoretical perspective for understanding the complexity inherent in the presence of the first wife in the couple relationship in a blended family after the loss of the children’s biological mother. According to SFT, the family system is composed of interrelated subsystems based on generation, gender, and role, which may consist of individuals or dyads (Minuchin, 1974). Studies conducted in recent decades (Rosenberg and Guttmann, 2001; Faber, 2004; Dupuis, 2010; Parent et al., 2014) show that the subsystems in the traditional two-parent family have now been joined by a variety of new subsystems that exist in other family models, such as non-biological parent and non-biological child, and non-biological siblings. Two additional subsystems that do not exist in the traditional family model emerged from the interviews in this study, namely, the partner’s couplehood with his first wife; and a triadic subsystem consisting of the new woman, her partner, and his deceased wife. In the eyes of the interviewees, these two subsystems exist side by side with, and impact, the couplehood they have built with their partner. This finding is in line with the results of previous studies conducted among widows and widowers (Moss and Moss, 1996; Silverman and Klass, 1996; Grinwald and Shabat, 1997; Brimhall and Engblom-Deglmann, 2011; Bokek-Cohen, 2014), showing that the continuing bond with the deceased partner influences the new couple relationship. Our participants reported feeling the presence of the subsystem of the partner and his first wife at varying intensity, becoming stronger primarily when they and their partner were having problems, at family events, and on dates associated with the deceased woman, such as the anniversary of her death and her birthday.

The participants’ sense that their partner’s relationship with his first wife exists alongside their couplehood relationship is consistent with the continuing bonds paradigm (Klass et al., 2014), whereby the bond with a deceased loved one persists throughout a bereaved individual’s life. It is manifested in various ways, including memories, keeping the deceased’s personal belongings, sensing the presence of the deceased, and identifying with the deceased by adopting their habits and values (Field et al., 2003). The women in this study referred to two major ways in which they felt their partner preserved his bond with his first wife, namely, keeping items associated with her and commemorations of her. The interviews revealed a complex picture of the women’s experience of their partner’s continuing bond with his deceased wife. The emotions they described ranged from positive to negative to ambivalent, with their response influenced primarily by the degree to which they perceived the bond to be central to their partner’s life. This might be explained in terms of the conceptualization proposed by Florczak and Lockie (2019), who characterize the continuing bond with the deceased spouse as either in the “foreground” or in the “background.” Bonds in the foreground color most of the bereaved spouse’s life and are central to it. Those in the background enable the individual to maintain the connection without dwelling on it and without it interfering with their desire to form new relationships. When the women in our study perceived their partner’s continuing bond with his first wife to be in the foreground, that is, when they described his difficulty parting from or storing her personal belongings or his ongoing preoccupation with her commemoration years into their relationship, they spoke of feeling insecure in their couplehood and that they were in competition with the deceased wife. Previous studies of widowhood (Moss and Moss, 1996; Brimhall and Engblom-Deglmann, 2011; Bokek-Cohen, 2014) reported similar findings, showing that when the continuing bond with the deceased spouse occupies a central place in the life of the partner, it may lead to difficulties in the new couplehood and a sense of insecurity in the new partner. Our findings indicate that these effects tend to lessen in intensity over time, coming to the fore primarily at family events and on dates associated with the first wife.

One of the most common reasons for the insecurity of the new partner is the bereaved partner’s reluctance to take off the wedding ring from their first marriage (Moss and Moss, 1996; Bokek-Cohen, 2014), an issue also raised in the interviews in this study. The wedding ring is a declaration of status, symbolizing continued commitment to the first marriage (Moss and Moss, 1996). When the symbolic act of removing the ring and thus letting go of the first marriage was not performed, our participants reported feeling their partner was in another relationship in addition to theirs. Bokek-Cohen (2014) similarly found that when widows did not cease to wear the ring from their previous marriage, their husbands felt their wives were married to two men.

The way in which our interviewees experienced their partner’s continuing bond with his first wife was also influenced by the manner in which he chose to describe her. When he idealized the deceased woman and related only to her positive qualities, the women felt inferior to her. Idealization of the deceased is a well-known phenomenon, emerging from studies of loss in general (O’Rourke, 2004; Hayes, 2016) and loss of a spouse in particular (Grinwald and Shabat, 1997; Brimhall and Engblom-Deglmann, 2011). Our findings are consistent with those of previous studies (Grinwald and Shabat, 1997; Brimhall and Engblom-Deglmann, 2011) showing that the tendency of a widower to idealize his first wife makes it difficult for his new partner to feel that she is appreciated and may cause her to compare the two relationships. In this context, Moss and Moss (1996) proposed the concept of “protective silence,” whereby both members of the couple refrain from referring to the deceased wife or the previous couplehood to avoid comparison or competition. This type of protective silence was only found among a small number of the participants in this study. On the contrary, most of the women noted their own need and that of their partner to present the first wife in their family life.

Another subsystem that emerged from the interviews in the wake of the partner’s continuing bond with his deceased wife was triadic, consisting of the woman, her partner, and his first wife. The concept of “Triangle” suggested by Bowen (1978) may provide a theoretical perspective for understanding this triadic subsystem. Bowen viewed the triangle as an integral part of human and family existence. Its purpose is to stabilize anxiety situations in intimate relationships between two people that arise primarily as a result of changes in the family life cycle, such as marriage, childbirth, children leaving home, divorce, illness, and death (Titelman, 2008). According to Bowen, the triangle is expressed in thoughts, fantasies, feelings, or behavior toward a third party outside the dyad (Klever, 2008), which serve to regulate the anxiety in the intimate relationship (Flaskas, 2012). Unlike Freud’s oedipal triangle, which relates to the triad of father-mother-child and is viewed as a stage in the child’s development (Hartke, 2016), the triangle Bowen proposed involves relations between all members of the family, both nuclear and extended, and lasts throughout their lives. In response to the controversy in the literature over whether or not a deceased family member can be seen as part of this triangle, Titelman (2008) distinguished between two types of triangles, namely, the emotional triangle and the partial mental construct triangle. The former consists only of living entities, while the latter is a mix of living and non-living entities, whether a deceased individual, an idea, a religion, or a philosophy. Our findings show that many of the women felt part of a triangle consisting of themselves, their partner, and his deceased wife and that this triangle constitutes an inherent component of their couplehood with a widowed partner. The feeling was strongest at times of family events and when memories in the family and previous couple’s space were aroused. A similar finding is reported by Moss and Moss (1996) on the basis of their study in 1981, which showed that the continuing bond with a deceased spouse creates a triadic construct that is an integral part of the new family structure when a widow or widower remarries. Kissman and Dane (2001) contend that the triadic subsystem that includes the first wife creates an obstacle to intimacy in a relationship with a widower. The findings of this study provide only partial support for this claim, as just a small number of participants reported that the triadic relationship hindered the creation of intimacy with their partner. On the other hand, many participants stated that their partner’s continuing bond with his first wife raised questions as to their own place and that of the deceased woman in the partner’s life, and caused them to wonder whether he could love two women at the same time.

An emotional bond with two women or two men within the framework of a monogamous relationship is liable to create cognitive dissonance (Morvan and O’Connor, 2017). A monogamous relationship, which is still the norm in Western countries, is defined as sexual and emotional relations with one romantic partner alone (Conley et al., 2013). Anderson (2010) distinguished between four types of monogamy, namely, physical monogamy, i.e., exclusivity of sexual relations with a partner; desirous monogamy, or exclusivity of desire and sexual fantasies toward a partner; social monogamy, i.e., the need to belong to the social category of a monogamous couple in view of social expectations that regard monogamy as the normative couple model; and emotional monogamy, or the need for an exclusive emotional couple relationship with a partner. The interviews in this study reveal that some of the participants felt that their couplehood was not grounded in emotional monogamy as a result of their partner’s continuing emotional bond with his deceased wife. As they saw it, their partner was conducting two parallel relationships.

A range of emotions were expressed here in the participants’ descriptions of coping with the question of their place and that of the first wife in the couple relationship, including contradictory feelings of understanding and encouraging the presencing of the deceased woman in the life of the family alongside the jealousy and even the sense that their partner was cheating on them. These findings shed light on the paradoxical reality with which women in a relationship with a widower must contend. As our interviewees portrayed it, this reality includes the partner’s continuing bond with his first wife alongside his love for them.

Future studies would benefit from exploring the couplehood experience of women who started a family with a widower with young children when they were divorced or widowed and had children of their own. Such investigations might provide insights into whether and how the woman’s family status shapes her experience of the new couplehood. Another interesting avenue for future research is to explore whether or not and in what ways having common children with their widowed partner impacts the couplehood experience of these women. Further research is also needed to investigate the role of both the widower’s children and their common children in shaping the couplehood experience of the new wife. It would also be of value to examine the experience of the widower in these relationships. In addition, longitudinal studies could increase our understanding of how these experiences change over time.

## 5. Conclusion

This study, which gives voice to women who started a family with a widower with young children, demonstrates the complexity and challenges with which they are forced to cope. The study views the couplehood experience of these women through three conceptual lenses, namely, the Continuing Bonds Paradigm (Klass et al., 2014), the notion of subsystems in Structural Family Therapy (Minuchin, 1974), and the Triangle as proposed by Bowen (1978). The findings expand our understanding of these theoretical concepts by revealing the link between them evidenced in the women’s narratives. Accordingly, the widower’s continuing bond with his deceased wife creates two subsystems that are inherent in the new relationship, namely, the previous subsystem of the partner and his first wife; and a triadic subsystem consisting of the woman, her partner, and his deceased wife. These two subsystems raise poignant questions regarding the place of the new woman in the couple’s relationship, indicating the complexity of her experience. The findings make a further contribution to the theoretical literature by showing that the nature of the triadic subsystem is influenced by the manner in which the woman perceives the quality and intensity of her partner’s continuing bond with his first wife and that this perception changes over time.

On the practical level, the study can aid professionals, particularly couple and family therapists, who offer support to blended families. Understanding the experience and unique challenges of women who start a family with a widower with young children can help them to tailor their counseling and the therapeutic process to the particular needs of these women. Our findings, which highlight the widowers’ continuing bond with their deceased wife, may also encourage professionals to address their loss within the therapeutic process and to help them embrace the present and nourish their new relationship.

## Data Availability Statement

The original contributions presented in the study are included in the article/supplementary material, further inquiries can be directed to the corresponding author.

## Ethics Statement

The studies involving human participants were reviewed and approved by the Ethics Committee of the Department of Social Work, Ben-Gurion University of the Negev. The patients/participants provided their written informed consent to participate in this study.

## Author Contributions

Both authors have made a substantial, direct, and intellectual contribution to the work, and approved it for publication.

## Conflict of Interest

The authors declare that the research was conducted in the absence of any commercial or financial relationships that could be construed as a potential conflict of interest.

## Publisher’s Note

All claims expressed in this article are solely those of the authors and do not necessarily represent those of their affiliated organizations, or those of the publisher, the editors and the reviewers. Any product that may be evaluated in this article, or claim that may be made by its manufacturer, is not guaranteed or endorsed by the publisher.

## Footnotes

1 The names and personal details of the participants have been changed to protect their privacy.

2 A ceremony that takes place on the thirteenth birthday of a Jewish boy.

3 A ceremony that takes place on the twelfth birthday of a Jewish girl.
